# Roles of ApoB-100 Gene Polymorphisms and the Risks of Gallstones and Gallbladder Cancer: A Meta-Analysis

**DOI:** 10.1371/journal.pone.0061456

**Published:** 2013-04-18

**Authors:** Yi Gong, Leida Zhang, Ping Bie, Huaizhi Wang

**Affiliations:** Institute of Hepatopancreatobiliary Surgery, Southwest Hospital, Third Military Medical University, Chongqing City, P. R. China; University of Bari & Consorzio Mario Negri Sud, Italy

## Abstract

**Background:**

Gallstones (GS) is the major manifestation of gallbladder disease, and is the most common risk factor for gallbladder cancer (GBC). Previous studies investigating the association between *ApoB-100* gene polymorphisms and the risks of GS and GBC have yielded conflicting results. Therefore, we performed a meta-analysis to clarify the effects of *ApoB-100* gene polymorphisms on the risks of GS and GBC.

**Methods:**

A computerized literature search was conducted to identify the relevant studies from PubMed and Embase. Fixed or random effects model was selected based on heterogeneity test. Publication bias was estimated using Begg’s funnel plots and Egger’s regression test.

**Results:**

A total of 10, 3, and 3 studies were included in the analyses of the association between *ApoB-100* XbaI, EcoRI, or insertion/deletion (ID) polymorphisms and the GS risks, respectively, while 3 studies were included in the analysis for the association between XbaI polymorphism and GBC risk. The combined results showed a significant association in Chinese (X+ *vs.* X−, OR = 2.37, 95%CI 1.52–3.70; X+X+/X+X- *vs.* X+X+, OR = 2.47, 95%CI 1.55–3.92), but not in Indians or Caucasians. Null association was observed between EcoRI or ID polymorphisms and GS risks. With regard to the association between XbaI polymorphism and GBC risk, a significant association was detected when GBC patients were compared with healthy persons and when GBC patients were compared with GS patients. A significant association was still detected when GBC patients (with GS) were compared with the GS patients (X+X+ *vs.* X-X−, OR = 0.33, 95%CI 0.12–0.90).

**Conclusion:**

The results of this meta-analysis suggest that the *ApoB-100* X+ allele might be associated with increased risk of GS in Chinese but not in other populations, while the *ApoB-100* X+X+ genotype might be associated with reduced risk of GBC. Further studies with larger sample sizes are needed to confirm these results.

## Introduction

Gallstone disease (GS) is the major manifestation of gallbladder diseases, and is one of the most common digestive disorders worldwide, especially in Western countries [1]. Although most GS are silent, 25% of gallstone carriers could develop symptoms and have to undergo cholecystectomy [2]. Accumulating evidences suggest that the pathogenesis of GS is multifactorial, and both environmental and genetic factors may be involved [3]. GS is on of the major risk factors for gallbladder cancer (GBC), which is the most common type of biliary tract cancer and the sixth most common form of digestive tract malignancy [4]. The association between GS and GBC has been known since 1861, and is supported by autopsy studies, screening surveys, and hospital-based case-control studies [5]. GS is classified into cholesterol stones and pigment stones according to their cholesterol content, and the cholesterol gallstone is more frequent than the pigment stones [6], [7]. The main cause of cholesterol gallstone formation is the supersaturation of bile with cholesterol in the gallbladder [6], thus the genes regulating the absorption, biosynthesis, and turnover of cholesterol might be associated with the risks of GS and GBC in individuals.

The liver is the major organ involved in the regulation of cholesterol metabolism. It could acquire cholesterol from plasma lipoproteins via endocytosis or selective cholesterol uptake mediated by the interactions of apolipoproteins with various cell surface molecules including low-density lipoprotein (LDL) receptor, LDL receptor- related protein, hepatic lipase, and scavenger receptor class B type 1 (SR-B1) [8]. Furthermore, the process of reverse cholesterol transport (RCT) allows peripheral cholesterol to be returned to liver, in which the high-density lipoprotein (HDL) plays a crucial role. Mature HDL can transfer cholesterol to the liver directly via SR-B1 or indirectly via cholesteryl ester transfer protein (CETP)-mediated transfer to ApoB- containing lipoproteins, with subsequent uptake by the liver via the LDLR [9]. After hepatic uptake, the cholesterol transported efficiently through the liver, and is ultimately secreted both as bile salts and unesterified cholesterol into the bile [8], [10]. The intrahepatic cholesterol trafficking involves different sterol binding/transfer proteins including sterol carrier protein-2 (SCP-2), which may efficiently deliver cholesterol to the canalicular region for secretion into the bile [8], [11]. The excretion of cholesterol from the liver is regulated by ATP-binding cassette half-transporters ABCG5 and ABCG8, which are typically expressed in hepatocytes, enterocytes and gallbladder epithelial cells [12], [13]. The transhepatic traffic of cholesterol from plasma lipoproteins into the bile is critical for overall cholesterol homeostasis and its alternations may lead to cholesterol GS formation. Polymorphisms of many genes such as ABCG5/ABCG8 and apolipoprotein E have been demonstrated to be associated with the risks of GS [14], [15].

Apolipoprotein B-100 (ApoB-100) is a key protein involved in lipid metabolism. It is the sole component of LDL particles and plays an important role in the homeostasis of LDL cholesterol in plasma [16]. APO-B100 is mainly synthesized in the liver and has an obligatory structural role in the formation of triglyceride-rich very low-density lipoprotein (VLDL) [17]. After triglyceride hydrolysis, most VLDL remnants are rapidly taken up by hepatocytes, but some are further metabolized to LDL that remains in the plasma with a half-life of approximately 20 hours [17].http://onlinelibrary.wiley.com/doi/10.1002/hep.20867/full - bib13 ApoB on the LDL particle acts as a ligand for LDL receptors in various cells throughout the body. High levels of ApoB can lead to plaques that cause vascular disease (atherosclerosis), leading to heart disease [18]. A recent study found that absence of ApoB expression in intestine reduced GS formation, probably by decreasing intestinal cholesterol uptake [19]. The human *ApoB-100* gene, located on 2p24-p23, is 43 kb in length with 81 bp signal sequence. Numerous polymorphisms have been identified in *ApoB-100*, among which the XbaI polymorphism (−2488C>T), a single base alteration in the exon 26, has been demonstrated to be associated with interindividual variability of lipid levels [20]. In addition, two other polymorphisms of *ApoB-100* gene, EcoRI (−4154G>A) and insertion/deletion (ID) polymorphisms have also been reported [21], [22].

To date, a series of epidemiological studies have been performed to investigate the relationship between *ApoB-100* gene polymorphisms and the risks of GS or the risks of GBC, but the obtained results were conflicting. Some of the studies supported strong associations between *ApoB-100* polymorphism and the diseases [23], [24], [25], [26], whereas others reported null association. Therefore, we performed a meta-analysis in order to provide a more accurate estimation of the association between the *ApoB-100* gene polymorphisms and the risks of GS and GBC.

## Methods

### Literature Search Strategy

A computerized literature search was performed for the available relevant studies from PubMed and Embase. The following keywords were jointly used: (“Apolipoprotein B” or “Apo B”) and (“polymorphism” or “mutation” or “variation”) and (“biliary stone” or “gallstone” or “cholelithiasis” or “gallbladder cancer” or “gallbladder carcinoma”). Additional studies were identified by a hand search of all the references of the retrieved articles. If more than one article was published using the same case series, only the study with largest sample size was selected. The literature search was updated to 30 November 2012.

### Inclusion Criteria and Data Extraction

The included studies must meet the following criteria: (1) evaluated the associations between *ApoB*-100 gene polymorphisms and the risks of GS or GBC, (2) case-control or cohort design, and (3) provided sufficient data for the calculation of odds ratios (ORs) with the corresponding 95% confidence interval (95%CI).

The following information was extracted from each study: (1) name of the first author, (2) publication year, (3) country of origin, (4) ethnicity of the study population, (5) source of the control subjects, (6) numbers of cases and controls, (7) gender and age of the enrolled subjects, and (8) number of genotypes in the cases and controls. The data were extracted independently by 2 investigators who reached a consensus on all of the items.

### Statistical Analysis

The associations of the *ApoB-100* gene polymorphisms and risks of GS or GBC were estimated by calculating the pooled ORs and 95%CI. The significance of the pooled effect size was determined by the *Z* test. Heterogeneity among the studies was assessed using the Q test and the *I^2^* test as performed in other studies [27], [28]. The DerSimonian and Laird random effects model was used as the pooling method when significant heterogeneity existed; otherwise, the Mantel-Haenszel fixed effect model was used [29]. Subgroup analyses were stratified by ethnicity. Influential analysis was undertaken by removing an individual study in each analysis to determine whether any single study could bias the overall estimate [30]. Begg’s funnel plots and Egger’s regression test were performed to assess the potential publication bias [31]. A probability of less than 0.05 was judged to be significant except for the *I* ^2^ statistic. The data analysis was performed using STATA version 10 (StataCorp LP, College Station, TX, USA).

## Results

### Characteristics of the Included Studies

**Figure 1:**
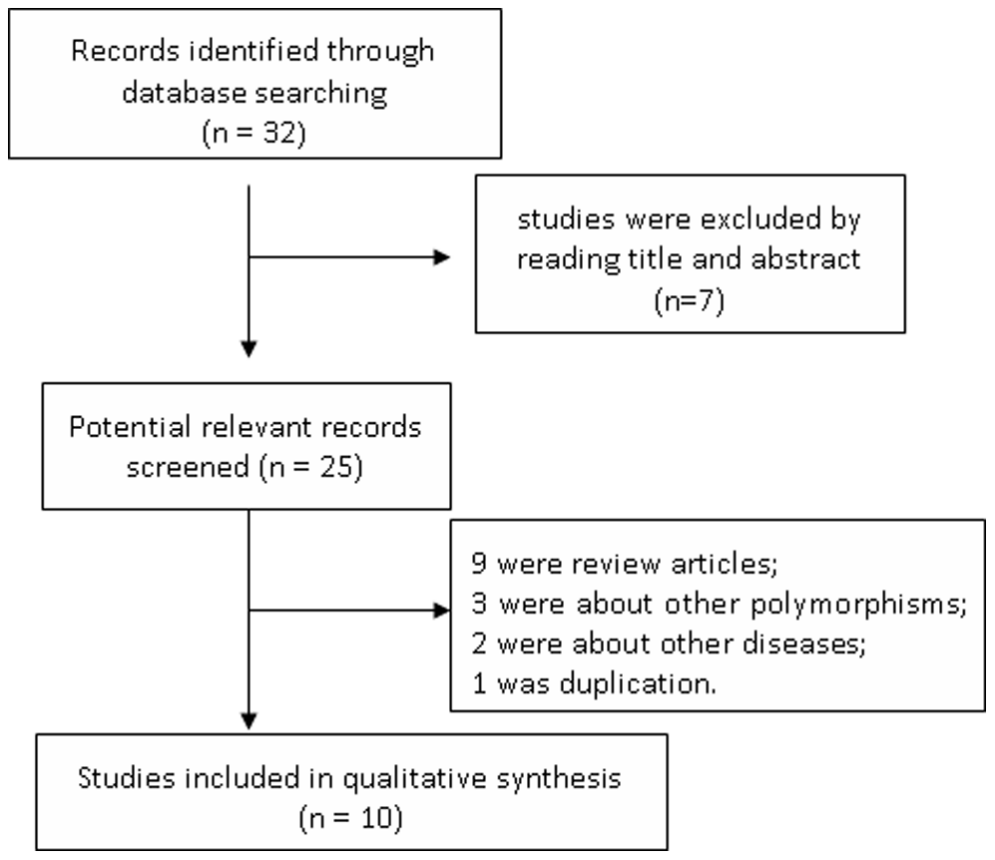
Flow diagram of study selection in this meta-analysis.

As depicted in **Figure 1**, 32 studies were identified through the database search. 7 obviously irrelevant studies were excluded after reading the titles and abstracts. 15 studies were further excluded as they were review articles, described unrelated gene polymorphisms or diseases, or were duplications. Therefore, 10 studies met the inclusion criteria and included in the meta-analysis. Among these studies, 3 studies investigated the XbaI and EcoRI polymorphisms [24], [26], [32], 3 studies investigated the XbaI and ID polymorphisms [33], [34], [35], while the remaining 4 studies only investigated the XbaI polymorphism [23], [25], [36], [37]. Thus, 10, 3, and 3 studies were finally included in the analyses of the association between the *ApoB-100* XbaI, EcoRI, ID polymorphisms and the risks of GS, respectively. Among these included studies, 3 studies were performed in Chinese, 4 in Indian subjects, 2 in Caucasians, 1 in Chile, and 1 in Mexico. For the association between the *ApoB-100* gene polymorphisms and the GBC risks, 3 studies were retrieved [23], [25], [34]. The studies by Pandey *et al.* [34] and by Singh *et al.* [25] enrolled GBC patients of 2 types (GBC with GS and GBC without GS), and thus were considered as 2 individual studies. All the included studies used blood samples for DNA extraction. Polymerase chain reaction-restriction fragment length polymorphism (PCR-REFLP) and TaqMan methods were used for genotyping. The detailed characteristics of the included studies are shown in **Table 1** and **Table 2**.

**Table 1:** Characteristics of individual studies for association between *ApoB-100* gene polymorphisms and risks of gall stones (GS).

Authors	Year	Country	Age^a^	Sex^b^	Type of GS	Source of control^c^	Genotypes distribution^d^						*P*_HWE_ e
							Case			control			
							11	12	22	11	12	22	
−**2488C>T (XbaI)**													
Baez	2010	Chile	42.7/45.8	Female	Na	HB	41	65	13	25	31	14	0.442
Sanchez-Cuen	2010	Mexico	51.9/51.7	86.1/86.1	Cholesterol	PB	41	51	9	34	50	17	0.849
Dixit	2008	India	44.7/44.0	32.2/36.0	Na	PB	117	83	6	177	127	16	0.261
Pandey	2007	India	46.9/42.2	33.7/46.1	Na	PB	98	68	9	127	94	11	0.220
Kurzawski	2007	Poland	61.9/63.9	24.6/47.0	Cholesterol	PB	63	129	48	61	122	34	0.036
Singh	2004	Inida	49.0/54.5	31.6/35.0	Na	PB	28	80	9	35	91	11	0.000
Jiang	2004	China	47.5/47.9	74.3/67.2	Na	PB	88	16	1	252	22	0	0.489
Tan	2003	China	52.2/51.3	27.4/30.5	Na	PB	84	22	0	94	11	0	0.571
Suo	1999	China	51.3/49.2	41.6/42.0	Cholesterol	HB	77	24	0	46	4	0	0.768
Juvonen	1995	UK	56.0/55.0	18.3/25.0	Cholesterol	PB	25	57	10	35	42	15	0.689
−**4154G>A (EcoRI)**													
Kurzawski	2007	Poland	61.9/63.9	24.6/47.0	Cholesterol	PB	172	63	5	128	74	15	0.344
Tan	2003	China	52.2/51.3	27.4/30.5	Na	PB	88	18	0	98	7	0	0.724
Juvonen	1995	UK	56.0/55.0	18.3/25.0	Cholesterol	PB	55	32	5	55	33	4	0.732
**insertion/deletion (ID)**													
Dixit	2008	India	44.7/44.0	32.2/36.0	Na	PB	148	53	6	165	84	6	0.214
Pandey	2007	India	46.9/42.2	33.7/46.1	Na	PB	122	43	7	153	76	3	0.055
Suo	1999	China	51.3/49.2	41.6/42.0	Cholesterol	HB	47	45	9	25	19	6	0.429

a the mean age of case and controls;

b the gender was shown as the percentage of the males;

c HB and PB referred to hospital-based controls and population-based controls, respectively;

d 11,12,22 represented X−X−, X−X+, X+X+ for XbaI polymorphism, GG, AG, AA for EcoRI polymorphism, II, ID, DD for insertion/deletion polymorphism, respectively;

e *p* for Hardy–Weinberg equilibrium test in controls;

“Na” means not available.

**Table 2:** Characteristics of individual studies for association between *ApoB-100* XbaI polymorphisms and risks of gall bladder cancer (GBC).

Authors	Year	Country	Cases	Source of control b	Case			control			*P*_HWE_ c
					X−X−	X+X−	X+X+	X−X−	X+X−	X+X+	
Baez	2010	Chile	GBC	HB	30	24	3	25	31	14	0.442
Pandey^a^	2007	India	GBC with GS	PB	42	24	1	127	94	11	0.220
Pandey^a^	2007	India	GBC without GS	PB	27	26	3	127	94	11	0.220
Singh^a^	2004	Inida	GBC with GS	PB	61	38	6	35	91	11	0.000
Singh^a^	2004	Inida	GBC without GS	PB	23	22	3	35	91	11	0.000

Abbreviations: GBC, gall bladder cancer; GS, gall stones;

a these two studies enrolled GBC patients of two types, i.e. GBC with GS and GBC without GS; and thus were considered as two individual studies;

b HB and PB referred to hospital-based controls and population-based controls, respectively;

c *p* for Hardy–Weinberg equilibrium test in controls.

### Quantitative Synthesis

**Figure 2:**
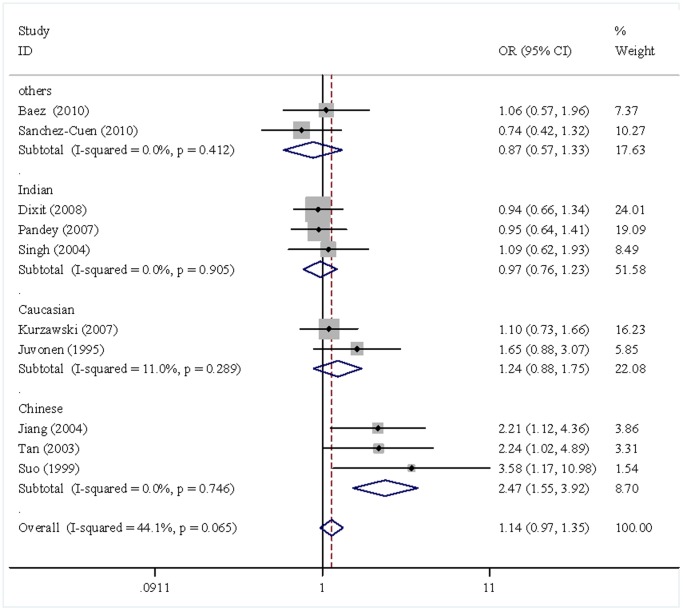
Meta-analysis of the *ApoB-100* XbaI polymorphisms and the risks of GS (genotype X+X+/X+X− *vs.* X−X−).

Overall, no significant association was observed between the *ApoB-100* XbaI polymorphism and the GS risk. However, the subgroup analyses by ethnicity showed a significant association in the Chinese (X+ *vs.* X−, OR = 2.37, 95%CI 1.52–3.70; X+X+/X+X− *vs.* X+X+, OR = 2.47, 95%CI 1.55–3.92) (**Figure 2**). In contrast, no significant association was detected in Indians and in Caucasians in any genetic model (**Table 3**). As GS is classified into cholesterol stones and pigment stones according to their cholesterol content, we then make subgroup analyses based on the types of GS. However, no significant association was found between *ApoB-100* XbaI polymorphism and the risks of cholesterol GS (X+ *vs.* X−, OR = 1.09, 95%CI 0.77–1.55; X+X+/X+X− *vs.* X+X+, OR = 1.28, 95%CI 0.78–2.10). With respect to the associations between the EcoRI polymorphism and the GS risk, no significant association was detected (A *vs.* G, OR = 1.05, 95%CI 0.50–2.23; AA/GA *vs.* GG, OR = 1.07, 95%CI = 0.48–2.42). Similarly, no significant association was observed between the ID polymorphism and the GS risks (D *vs.* I, OR = 0.88, 95%CI 0.71–1.11; DD/DI *vs.* II, OR = 0.81, 95%CI 0.62–1.06). As limited studies were included in the analyses of the EcoRI and ID polymorphisms and the risks of GS, we did not perform subgroup analysis.

**Table 3:** Summary of ORs for various contrasts on the association between *ApoB-100* gene polymorphisms and risks of gall stones (GS).

	Sub-group	Test of association			Test of heterogeneity	
		OR	95%CI	Model^a^	*I* ^2^ (%)	*p value* b
−**2488C>T (XbaI)**						
X+ *vs.* X−	All	1.09	0.90–1.31	REM	51.0	0.031
	China	2.37	1.52–3.70	FEM	0.0	0.800
	India	0.96	0.80–1.16	FEM	0.0	0.861
	Caucasian	1.13	0.91–1.41	FEM	0.0	0.961
X+X+ *vs.* X−X−	All	0.90	0.66–1.23	FEM	16.5	0.300
	China	Na	Na	FEM	Na	Na
	India	0.84	0.49–1.46	FEM	0.0	0.599
	Caucasian	1.24	0.76–2.01	FEM	0.0	0.498
X+X− *vs.* X−X−	All	1.18	1.00–1.40	FEM	39.6	0.094
	China	2.41	1.51–3.83	FEM	0.0	0.709
	India	0.99	0.77–1.26	FEM	0.0	0.908
	Caucasian	1.24	0.87–1.77	FEM	58.6	0.120
X+X+/X+X− *vs.*X−X−	All	1.14	0.97–1.35	FEM	44.1	0.065
	China	2.47	1.55–3.92	FEM	0.0	0.746
	India	0.97	0.76–1.23	FEM	0.0	0.905
	Caucasian	1.24	0.88–1.75	FEM	11.0	0.289
−**4154G>A (EcoRI)**						
A *vs.* G		1.05	0.50–2.23	REM	83.4	0.002
AA *vs.* GG		0.52	0.11–2.54	REM	70.7	0.065
GA *vs.* GG		1.10	0.51–2.33	REM	77.6	0.012
AA/GA *vs.* GG		1.07	0.48–2.42	REM	81.7	0.004
**insertion/deletion**						
D *vs.* I		0.88	0.71–1.11	FEM	0.0	0.740
DD *vs.* II		1.29	0.65–2.57	FEM	5.2	0.348
DI *vs.* II		0.77	0.58–1.01	FEM	4.7	0.350
DD/DI *vs.* II		0.81	0.62–1.06	FEM	0.0	0.525

a Model, statistical model; FEM, fixed effect model; REM, random effect model.

b *p* value for heterogeneity based on Q test;

Na, not available.

**Figure 3:**
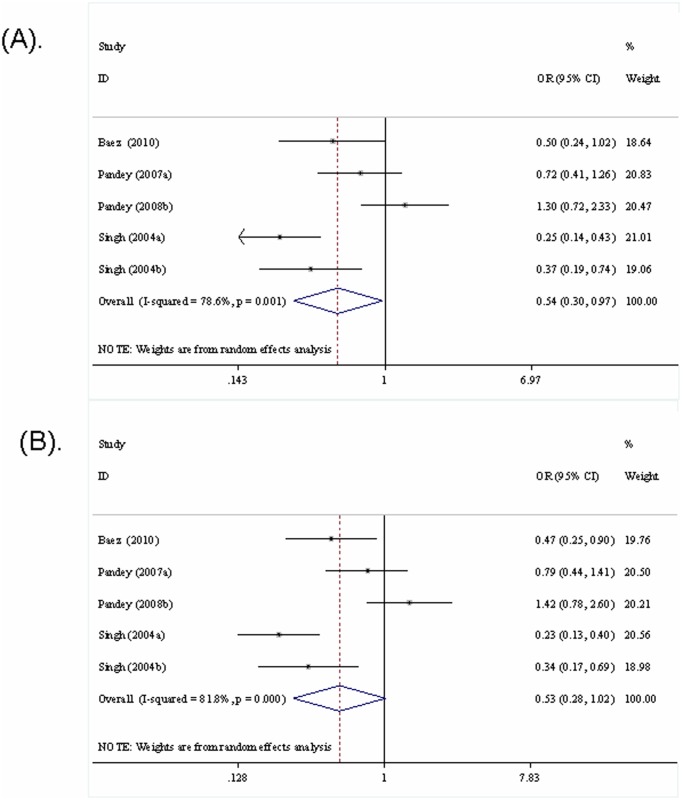
Meta-analysis of the *ApoB-100* XbaI polymorphism and the risks of GBC (genotype X+X+/X+X− *vs.* X−X−).

The results of the pooled analysis for the association between the *ApoB-100* XbaI polymorphism and the GBC risk were shown in **Table 4** **and** **Figure 3**. The comparisons were made between GBC patients and healthy persons, and between GBC patients and GS patients, respectively. When all the studies were combined, significant associations were observed between the XbaI polymorphism and GBC risk (X+ *vs.* X−, OR = 0.64, 95%CI 0.45–0.92; X+X+/X+X− *vs.* X−X−, OR = 0.54, 95%CI 0.30–0.97) when GBC patients were compared with healthy persons. However, the subgroup analysis based on the types of GBC (with or without GS) showed no significant association between the XbaI polymorphism and GBC risk when GBC patients without GS were compared with healthy persons (X+ *vs.* X−, OR = 0.85, 95%CI 0.42–1.70; X+X+ *vs.* X−X−, OR = 0.71, 95%CI 0.27–1.84). For the comparisons between the GBC patients and GS patients, significant association was also detected for the homozygous comparison (X+X+ *vs.* X−X−, OR = 0.45, 95%CI 0.24–0.85). Furthermore, significant association could still be detected when the GBC patients with GS were compared with the GS patients (X+X+ *vs.* X−X−, OR = 0.33, 95%CI 0.12–0.90).

**Table 4:** Summary of ORs for various contrasts on the association between *ApoB-100* gene polymorphisms and risks of gall bladder cancer (GBC).

	Contrasts	Test of association				Test of heterogeneity	
		OR	95%CI	*P* value	Model^a^	*I* ^2^ (%)	*p value* b
**GBC ** ***vs.*** ** healthy person**							
X+ *vs.* X−	All	0.64	0.45–0.92	0.016	REM	65.7	0.020
	GBC without GS	0.85	0.42–1.70	0.638	REM	76.5	0.039
X+X+ *vs.* X−X−	All	0.37	0.20–0.69	0.001	FEM	12.9	0.332
	GBC without GS	0.71	0.27–1.84	0.477	FEM	24.8	0.249
X+X− *vs.*X−X−	All	0.56	0.31–1.04	0.068	REM	79.0	0.001
	GBC without GS	0.70	0.20–2.42	0.574	REM	86.1	0.007
X+X+/X+X− *vs.* X−X−	All	0.54	0.30–0.97	0.038	REM	78.6	0.001
	GBC without GS	0.71	0.21–2.40	0.576	REM	86.5	0.007
**GBC ** ***vs.*** ** GS**							
X+ *vs.* X−	All	0.68	0.46–1.00	0.050	REM	69.6	0.011
	GBC with GS	0.58	0.32–1.04	0.069	REM	70.6	0.065
X+X+ *vs.* X−X−	All	0.45	0.24–0.85	0.013	FEM	7.2	0.366
	GBC with GS	0.33	0.12–0.90	0.030	FEM	0.0	0.845
X+X− *vs.* X−X−	All	0.53	0.28–1.03	0.062	REM	81.5	0.000
	GBC with GS	0.42	0.12–1.56	0.196	REM	89.7	0.002
X+X+/X+X− *vs.* X−X−	All	0.53	0.28–1.02	0.056	REM	81.8	0.000
	GBC with GS	0.42	0.13–1.43	0.167	REM	88.8	0.003

a Model, statistical model; FEM, fixed effect model; REM, random effect model.

b *p* value for heterogeneity based on Q test;

Abbreviations: GS, gallstone; GBC, gallbladder cancer.

### Heterogeneity Source and Sensitivity Analysis

No significant heterogeneity was detected in the analysis between the *ApoB-100* ID polymorphism and GS risk. A moderate heterogeneity was detected in the analysis of the association between the *ApoB-100* XbaI polymorphism and GS risk (*I^2^<*50% in most comparisons). The heterogeneity was significantly reduced in the subgroup analyses stratified by ethnicity, suggesting that ethnicity might be a major contributor to the between-study heterogeneity (**Table 3**). However, a significant between-study heterogeneity was observed in the analyses between the *ApoB-100* EcoRI polymorphism and GS risk and between the *ApoB-100* XbaI polymorphism and GBC risk (*I^2^>*50% in most comparisons). Although we did not perform the subgroup analysis due to the limitations of the studies included, ethnicity might also be a major contributor to the heterogeneity. Sensitivity analysis was performed by the sequential omission of individual studies in every comparison, and the data showed that no study significantly influenced the pooled effects based on the omission of any single study. The exclusion of the studies that deviated from HWE did not change the results significantly.

### Publication Bias

Potential publication bias was detected by Egger’s test in the comparisons of X+X+/X+X− *vs.* X−X−, D *vs.* I, DI *vs.* II, and DD/DI *vs.* II in the analyses between the *ApoB-100* gene polymorphisms and the risks of GS. No significant publication bias was detected in any other comparison (P_value_ *>*0.05 in both Egger’s regression and Begg’s rank correlation tests).

## Discussion

GS is a common digestive disease worldwide and is also a major risk factor for GBC [5]. Accumulating evidence has suggested that both genetic and environmental factors contribute to the onset of GS and GBC. A variety of epidemiological studies have been performed to explore the associations between several candidate genes and the risks of GS and GBC. Among these candidate genes, *ApoB-100* is of particular interest, as it is the major protein component of LDL [16]. In fact, some studies have suggested that individuals with the X+X+ genotype have significantly higher serum total cholesterol, LDL, and Apo-B levels compared with those with the wild-type X−X− genotype [38]. Thus, this *ApoB-100* variant may be related to a higher incidence of GS and GBC. Unfortunately, previous epidemiological studies investigating the associations between *ApoB-100* gene polymorphisms and the risks of GS/GBC have yielded conflicting results. The discrepancy may be attributed to multiple factors, such as the ethnicity of the population, the type of GS, and the sample size. Thus, a timely meta-analysis is necessary.

In this study, we performed a comprehensive literature search and included a total of 10, 3, and 3 studies for the analyses between the *ApoB-100* XbaI, EcoRI, and ID polymorphisms, and the risks of GS, respectively. The combined results showed a significant association in Chinese (X+ *vs.* X−, OR = 2.37, 95%CI 1.52–3.70; X+X+/X+X− *vs.* X−X−, OR = 2.47, 95%CI 1.55–3.92) but not in Indians or Caucasians (**Table 3**). Neither of the other polymorphisms, EcoRI or ID, was significantly associated with the GS risks. With regard to the associations between the *ApoB-100* XbaI polymorphism and the GBC risk, the pooled results of all the studies showed a significant association when GBC patients were compared with healthy persons and when GBC patients were compared with GS patients. However, the subgroup analysis showed no significant association between the XbaI polymorphism and GBC risk when GBC patients without GS were compared with healthy persons, while a significant association could be still detected when GBC patients with GS were compared with GS patients (X+X+ *vs.* X−X−, OR = 0.33, 95%CI 0.12–0.90). These data suggest that the *ApoB-100* X+ allele might be associated with an increased GS risk in Chinese, whereas the X+X+ genotype might be associated with a reduced risk of GBC.

The X+X+ genotype has been reported to be associated with higher serum levels of LDL compared with the X−X− genotype [39], [40]. Therefore, the speculation that X+X+ might be associated with increased risks of GS is reasonable. The results of this meta-analysis showed that the X+ allele was associated with an increased risk of GS in Chinese, but not in Indians and Caucasians. The discrepant effects may be related with several factors. In Chinese, the X+ allele frequency was about 4.42%, whereas it was approximately 36.8% in other populations. Therefore, the different results might be, at least partially, attributed to the ethnicity-related distribution of the X+ allele frequency. Furthermore, GS has bee reported to be more prevalent in countries consuming a western diet [41]. Thus, the different dietary habit might be another contributor for the discrepancy in the association between *ApoB-100* XbaI polymorphisms and the GS risks in different populations, as GS is the results of the interaction between genetic and environmental factors [3]. Considering that the 3 studies enrolled for the subgroup analyses for Chinese in this study were all performed about 10 years ago, and there has been a considerable change with adaptation of western diet for Chinese in recent years, a well-designed epidemiological study is necessary to investigate whether *ApoB-100* XbaI polymorphism could still influence the risks of GS for Chinese. For GBC, the results of the current meta-analysis showed that X+ might be related to a reduced risk of GBC. It is difficult to explain by what mechanisms the X+ allele decreases the risks of GBC.

So far, most genetic association studies about the gene polymorphisms and GS risks are in case-control design and have not been replicated in larger population cohorts and other ethnic groups [3]. The only genome-wide association study (GWAS) by Buch *et al.* scanned >500,000 SNPs in 280 individuals with GS and 360 controls, and identified the ABCG8 as a susceptibility factor for human GS [14]. At present, conflicting results about the association between *ApoB-100* gene polymorphisms and the risks of GS were obtained from case-control studies performed in different populations. Furthermore, limited experimental studies have focused on the roles of ApoB-100 in the pathogenesis of GS. In one study, Zhao *et al.* found that high cholesterol diet fed rabbits showed higher concentrations of plasma ApoB-100, which might enhance the secretion of biliary cholesterol into bile, ant thus facilitate the formation of GS [42]. However, in another study, *ApoB-100* gene deficiency did not reduce biliary cholesterol secretion and cholelithogenesis, while the mice deficiency of *ApoB-48* displayed significantly lower secretion rates of biliary cholesterol and significant decreases in prevalence rates, numbers, and sizes of GS [19]. Therefore, the roles of ApoB-100 in the pathogenesis of GS remains to be further studied.

Some limitations of this meta-analysis should be noted in interpreting the results. First, the present meta-analysis was based on unadjusted effect estimates and CIs because most studies did not provide the adjusted ORs and 95%CI. Second, GS/GBC are multifactor diseases, however, the interaction of gene-gene and gene-environment were not addressed in the current study, and thus the potential roles of the above gene polymorphisms might be masked or magnified by other gene-gene/gene-environment interactions. Third, the possibility of a selection bias cannot be completely excluded because only published studies were included. Although we searched multiple databases, we may have still failed to include some papers.

In conclusion, the current meta-analysis suggests that the *ApoB-100* X+ allele might be associated with increased risk of GS in Chinese but not in other populations, while the *ApoB-100* X+X+ genotype might be associated with reduced risk of GBC. Further studies with larger sample sizes are needed to confirm these results.

## Supporting Information

PRISMA Checklist S1 (DOC)
